# Supplementary material for: Ion solvation as a predictor of lanthanide adsorption structures and energetics in alumina nanopores
Source: Commun Chem. 2023 Aug 22;6:172. doi: 10.1038/s42004-023-00978-3 (PMC10444809; doi:10.1038/s42004-023-00978-3)
Supplement: Supplementary file 2 — Supplementary Information [file 42004_2023_978_MOESM2_ESM.pdf]

Supporting Information

Ion solvation as a predictor of lanthanide adsorption structures and energetics in alumina nanopores

Anastasia G. Ilgen[*1], Nadine Kabengi[2], Jacob G. Smith[1], and Kadie M.M. Sanchez[1]*

[1] Geochemistry Department, Sandia National Laboratories, 1515 Eubank Boulevard SE, Albuquerque, NM 87123, United States.

[2] Department of Geosciences, Georgia State University, 24 Peachtree Center Avenue NE Atlanta, GA 30303, United States

*Corresponding author: agilgen@sandia.gov

Summary of data used for thermodynamic calculations

Table S1 shows Nd, Tb, and Lu concentrations measured using inductively coupled plasma mass spectrometry (ICP-MS) in batch adsorption samples equilibrated at different temperatures. Calculated surface-area-normalized lanthanide uptake (Kd) and ln(Kd) values are shown.

Table S1. Adsorbates (Nd, Tb, or Lu) are shown in sample names. Al_2O_3 denotes aluminum oxide with 4 nm pores, and "Cor" denotes corundum samples.

Sample Id	Temp, ◦C	C1 (ppb)	C2 (ppb)	Uptake (mol/m^2)	Ln (Kd)
Al_2O_3-Nd-50-65	65	48536	24435	0.000498784	-7.60334
Al_2O_3-Nd-50-55	55	48536	28825	0.000407942	-7.80439
Al_2O_3-Nd-50-45	45	48536	34131	0.000298128	-8.11799
Al_2O_3-Nd-50-35	35	48536	34489	0.00029071	-8.14318
Al_2O_3-Nd-50-25	25	48536	37541	0.000227564	-8.38808
Cor-Nd-50-65	65	48536	40758	0.036710145	-3.3047
Cor-Nd-50-55	55	48536	41116	0.035020679	-3.35182
Cor-Nd-50-45	45	48536	43191	0.025229794	-3.67973
Cor-Nd-50-35	35	48536	46321	0.010455668	-4.56061
Cor-Nd-50-25	25	48536	49780	0	
Al_2O_3-Lu-50-65	65	67275	16721	0.000862492	-7.05568
Al_2O_3-Lu-50-55	55	67275	24024	0.000737885	-7.21172
Al_2O_3-Lu-50-45	45	67275	37692	0.000504707	-7.59153
Al_2O_3-Lu-50-35	35	67275	46253	0.000358649	-7.93317
Al_2O_3-Lu-50-25	25	67275	48328	0.00032325	-8.03708
Cor-Lu-50-65	65	67275	35946	0.121891088	-2.10463
Cor-Lu-50-55	55	67275	45472	0.084826434	-2.46715
Cor-Lu-50-45	45	67275	51043	0.063152118	-2.76221
Cor-Lu-50-35	35	67275	55677	0.045122078	-3.09838
Cor-Lu-50-25	25	67275	63523	0.014595659	-4.22703
Al_2O_3-Tb-50-65	65	126341	45382	0.001520646	-6.48862
Al_2O_3-Tb-50-55	55	126341	55823	0.001324545	-6.62669
Al_2O_3-Tb-50-45	45	126341	57263	0.001297496	-6.64732
Al_2O_3-Tb-50-35	35	126341	60652	0.001233838	-6.69763
Al_2O_3-Tb-50-25	25	126341	72333	0.001014437	-6.89342
Cor-Tb-50-65	65	126341	68570	0.247456965	-1.39652
Cor-Tb-50-55	55	126341	76567	0.213200779	-1.54552
Cor-Tb-50-45	45	126341	75277	0.218727635	-1.51993
Cor-Tb-50-35	35	126341	81214	0.193295717	-1.64353
Cor-Tb-50-25	25	126341	83819	0.182140655	-1.70298
Al_2O_3-Nd-5-65	65	4854	162	97.096×10^{-6}	-9.24
Al_2O_3-Nd-5-55	55	4854	988	80.005×10^{-6}	-9.43
Al_2O_3-Nd-5-45	45	4854	1450	70.439×10^{-6}	-9.56

Al_2O_3-Nd-5-35	35	4854	2530	48.081×10^{-6}	-9.94
Al_2O_3-Nd-5-25	25	4854	2703	44.511×10^{-6}	-10.02
Cor-Nd-5-65	65	4854	97	22447.878×10^{-6}	-3.80
Cor-Nd-5-55	55	4854	1660	21982.239×10^{-6}	-4.19
Cor-Nd-5-45	45	4854	3069	8423.617×10^{-6}	-4.78
Cor-Nd-5-35	35	4854	4383	2223.102×10^{-6}	-6.11
Cor-Nd-5-25	25	4854	4383	2220.260×10^{-6}	-6.11
Al_2O_3-Lu-5-65	65	6727	16	114.499×10^{-6}	-9.07
Al_2O_3-Lu-5-55	55	6727	136	112.451×10^{-6}	-9.09
Al_2O_3-Lu-5-45	45	6727	731	92.237×10^{-6}	-9.19
Al_2O_3-Lu-5-35	35	6727	697	102.880×10^{-6}	-9.18
Al_2O_3-Lu-5-25	25	6727	1156	95.049×10^{-6}	-9.26
Cor-Lu-5-65	65	6727	200	25397.789×10^{-6}	-3.67
Cor-Lu-5-55	55	6727	1923	18692.093×10^{-6}	-3.98
Cor-Lu-5-45	45	6727	3834	11256.789×10^{-6}	-4.49
Cor-Lu-5-35	35	6727	4835	7361.190×10^{-6}	-4.91
Cor-Lu-5-25	25	6727	5401	6258.480×10^{-6}	-5.07
Al_2O_3-Tb-5-65	65	12634	96	235.510×10^{-6}	-8.35
Al_2O_3-Tb-5-55	55	12634	954	219.380×10^{-6}	-8.42
Al_2O_3-Tb-5-45	45	12634	1415	0.184×10^{-6}	-8.46
Al_2O_3-Tb-5-35	35	12634	2601	188.457×10^{-6}	-8.58
Al_2O_3-Tb-5-25	25	12634	3748	166.914×10^{-6}	-8.70
Cor-Tb-5-65	65	12634	2419	43753.514×10^{-6}	-3.13
Cor-Tb-5-55	55	12634	5244	31655.903×10^{-6}	-3.45
Cor-Tb-5-45	45	12634	6229	27434.096×10^{-6}	-3.60
Cor-Tb-5-35	35	12634	6915	24498.201×10^{-6}	-3.71
Cor-Tb-5-25	25	12634	7413	22365.455×10^{-6}	-3.80

X-ray absorption fine structure spectroscopy data plotted in k-space for all lanthanide adsorption samples

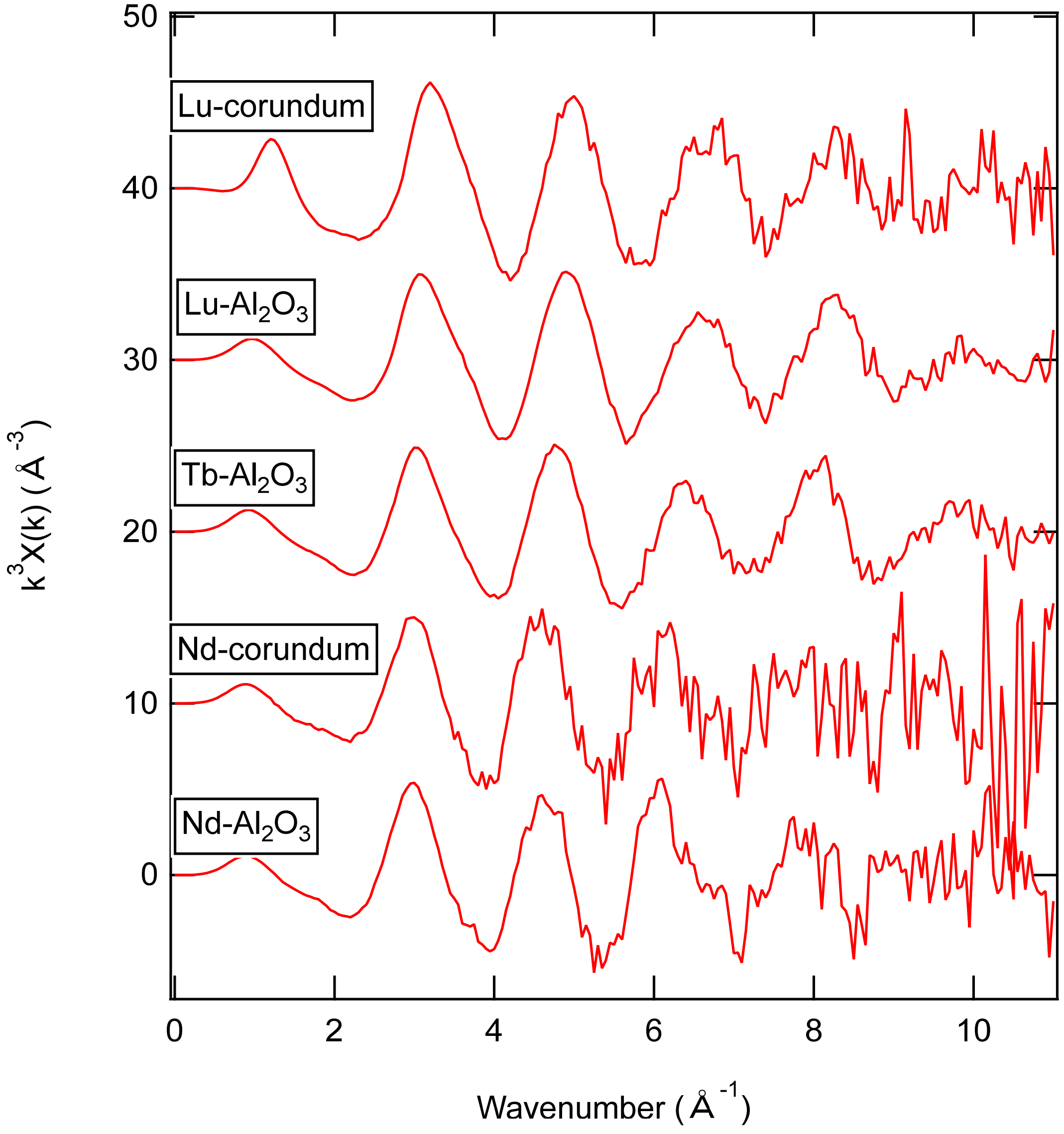

Figure S1. All X-ray absorption fine structure spectroscopy data collected for Nd, Tb and Lu at the Advanced Photon Source.

X-ray absorption fine structure spectroscopy data and corresponding fits for terbium

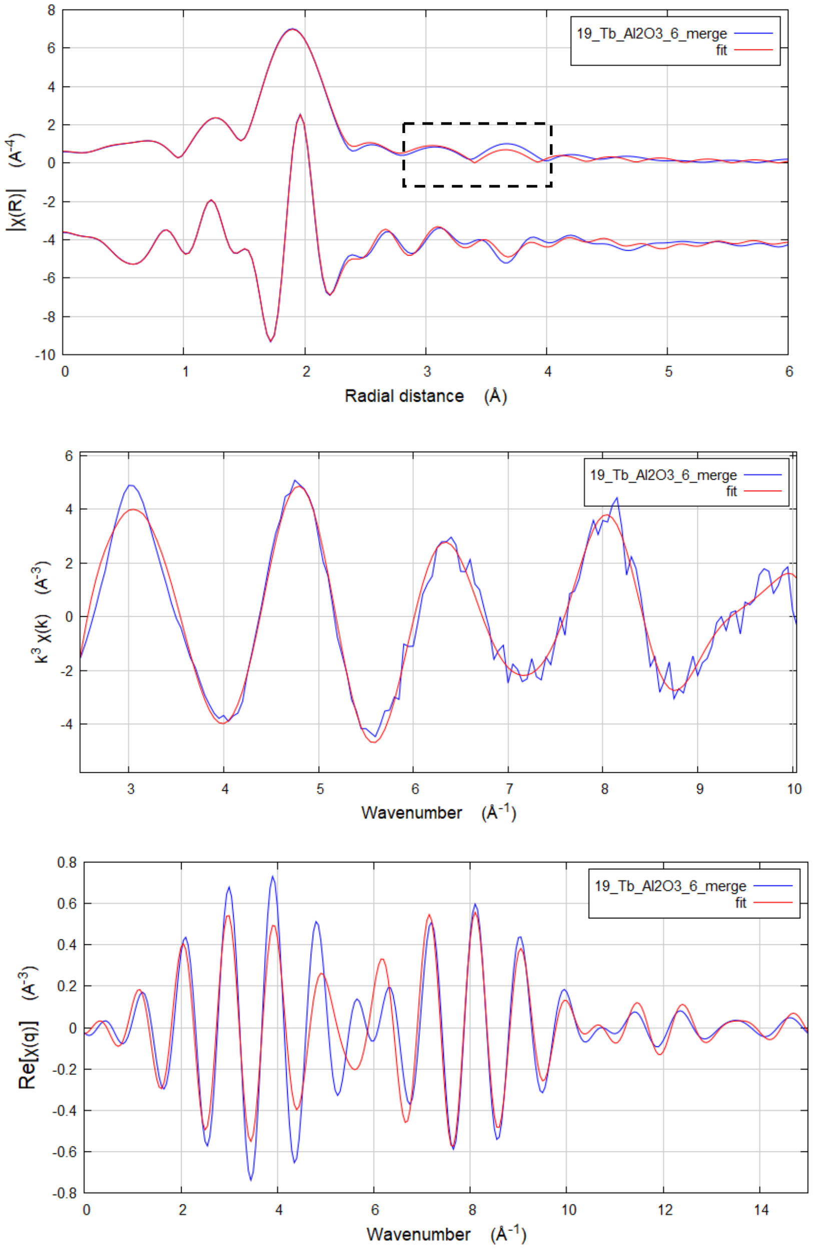

Figure S2. X-ray absorption fine structure spectroscopy data collected for Tb (blue) and fits (red). The bottom panel shows backward Fourier transformed XAFS data (blue) and fit (red) for the R-range from 2.8-4.0 (this R-range is shown in a dashed box in the top panel).
